# Chronic idiopathic myopathy in Icelandic horses: A case series

**DOI:** 10.1111/evj.14519

**Published:** 2025-04-24

**Authors:** Sanni Hansen, Charlotte Hopster‐Iversen, Lise Berg, Julie Fjeldborg, Claire Massey, Richard J. Piercy, Helena Carstensen

**Affiliations:** ^1^ Department of Veterinary Clinical Sciences Faculty of Health and Medical Sciences, University of Copenhagen Taastrup Denmark; ^2^ Comparative Neuromuscular Diseases Laboratory Royal Veterinary College London UK

**Keywords:** creatine kinase, horse, lameness, neuromuscular disease, poor performance, stumbling

## Abstract

**Background:**

Exertional myopathies are recognised as a cause of poor performance in equines. In Icelandic horses presenting reduced performance and/or multi‐limb lameness, no specific myopathy has been identified.

**Objectives:**

To characterise the clinical presentation and histopathological findings in muscle biopsy samples from Icelandic horses demonstrating poor performance.

**Study Design:**

Case series.

**Methods:**

Phenotypic characteristics, clinical examination and diagnoses of 17 Icelandic horses were studied. All horses had a resting serum creatine kinase (CK) and aspartate transaminase activities measured, and CK activities 4–6 and 24 h post‐exercise were measured in some horses. The semimembranosus muscle was biopsied in all horses and used to categorise horses into two groups: those with histopathological evidence of chronic idiopathic myopathy and those with normal biopsy findings.

**Results:**

Twelve horses displayed non‐specific histological features of muscle degeneration and regeneration consistent with a myopathy. The affected horses had significantly increased CK activities after exercise (median 1637 U/L, IQR 861–2480 U/L) compared with the group with histologically normal muscle (median 234 U/L, IQR 211–457 U/L, *p* = 0.02). Phenotypic traits, such as tachypnoea, fatigue, stumbling and reluctance to tölt or canter, were present in both groups.

**Main Limitations:**

The small sample size and absence of a control group with normal performance.

**Conclusions:**

This case series suggests the presence of a novel myopathy in Icelandic horses. In horses of this breed, exhibiting decreased performance, multi‐limb lameness and/or increased serum CK activity at rest or post‐exercise, an underlying myopathy should be considered.

## 1 INTRODUCTION

Stiffness, stumbling and reluctance to move forward are common complaints in poorly performing horses with potential etiologies spanning metabolic, orthopaedic and neuromuscular origins. Exertional muscle disorders in particular, which cause varying degrees of muscle weakness and pain, should be considered as differential diagnoses in horses with nonspecific signs of poor performance. Exertional myopathies with suspected or recognised genetic origins in different equine breeds have variable clinical presentations, aetiologies, pathophysiological mechanisms and severities.^1^, ^2^ Indeed, myopathies have been implicated in 17% of cases involving otherwise sound but poorly performing Standardbred racehorses.^3^ In Thoroughbred racehorses, the prevalence of exertional rhabdomyolysis has been reported to be between 4.9% to 6.7%,^4^, ^5^ while the overall prevalence of exertional rhabdomyolysis across different breeds in Australia is 1.9%, with significantly higher prevalence observed in exercising horses compared with non‐exercising horses.^6^ To our knowledge, reports of myopathies in Icelandic horses remain sparse.^7^

The Icelandic horse has special gaits and is an increasingly popular leisure horse and competitive athlete. Icelandic horses stand at about 130–150 cm at the withers, but despite their relatively small height, they are often ridden by adults. Several issues concerning their performance and weight carrying capacity have been reported.^8^, ^9^, ^10^, ^11^ Among those, a pilot study revealed considerable low‐grade lameness among Icelandic horses,^12^ although mild lameness is difficult for horse owners to recognise.^13^ The Icelandic breed poses a clinical challenge, in particular when seeking to differentiate between myopathic signs and more common causes of (multi‐limb) musculoskeletal lameness, since some Icelandic horses struggle to perform a regular two‐beat trot and may only be able to perform the four‐beat gait tölt.^8^

This study aimed to characterise the clinical presentation of Icelandic horses exhibiting poor performance with and without increased muscle enzyme activity and histopathological findings in muscle biopsy samples.

## 2 MATERIALS AND METHODS

### 2.1 Horses

Seventeen Icelandic horses, 15 geldings and two mares (mean age 11 years [range 6–19 years]) were included in this case series. Included horses were presented at the Large Animal Teaching Hospital, University of Copenhagen with a history of poor performance and the semimembranosus muscle was biopsied for histopathological evaluation. Phenotypic characteristics, history and clinical examination findings were noted, and haematology and serum biochemistry were evaluated, including baseline measurements of serum creatine kinase (CK) and aspartate transaminase (AST) activity. In a subset of horses, CK activities at 4–6 h and 24 h after a non‐standardised exercise test were available. The exercise tests were not identical, but all included between 15 and 30 min of lungeing or ridden trot, tölt and/or canter.

### 2.2 Initial diagnostics

As all horses were presented with a history of poor performance, additional diagnostic workup was applied to all cases based on the decisions of the examining veterinarians, or the owner asked for a poor performance workup package including a ridden evaluation equipped with over‐ground endoscopy and ECG, a resting endoscopy including bronchoalveolar lavage (BAL) and echocardiography. All but two of the included horses had additional diagnoses (Table 1). A diagnosis of mild–moderate equine asthma (MMEA) was based on BAL cytology and clinical presentation, according to reference values proposed by the consensus statement,^14^ as MMEA is a differential diagnosis for all horses presented with poor performance, or a clinical presentation of occasional cough or increased respiratory rate. The diagnosis of dorsal displacement of the soft palate was based on overground endoscopic examination. Lameness was diagnosed following an orthopaedic examination applying both subjective and objective lameness (The Equinosis Q with Lameness Locator software) evaluation. For all lameness cases (6 horses) regional anaesthesia was performed to localise the lameness. Gastric ulcers were diagnosed by gastroscopy after 16 h of feed withdrawal and hepatic disease was diagnosed by serum biochemistry (increased glutamate dehydrogenase, gamma‐glutamyl transpeptidase, AST) and liver biopsy (mild portal fibrosis).

**TABLE 1:** Creatine kinase (CK) and aspartate transaminase (AST) enzyme values in U/L at baseline and after exercise.

	CK baseline	AST baseline	CK 4‐6 h	CK 24 h
Group M (*n* = 12)	462a (246–1550)	570 (432–711)	1637ab (861–2480) (*n* = 10)	1875c (435–2691) (*n* = 6)
Group N (*n* = 5)	370 (200–406)	400 (359–675)	234b (211–457) (*n* = 3)	262c (186–397) (*n* = 3)

*Note*: Results are displayed as median, 25th and 75th quartiles for horses with histopathological changes in their muscle biopsies (Group M) and horses with histologically normal muscle biopsies (Group N). Medians with the same letter (a–c), both within row and column, indicate significant differences between groups.

### 2.3 Muscle biopsy samples

The semimembranosus muscle was biopsied using an open method as previously described.^15^ A fresh sample was enclosed in a plastic pot and packaged with icepacks and shipped (within 24 h) to the Comparative Neuromuscular Diseases Laboratory, Royal Veterinary College, London UK for further processing and analysis. Fresh samples (approximately 5 mm diameter) were trimmed and frozen in isopentane‐cooled liquid nitrogen on cork discs using standard techniques.^16^ Thereafter, sections were cryosectioned at 8 μm (Bright) and stained with a panel of histological and histochemical stains that included haematoxylin and eosin, Gomori trichrome, Periodic Acid Schiff (PAS) with and without prior diastase digestion (16 μm), Oil red O, succinate dehydrogenase, nicotinamide adenine dinucleotide (NADH) and cytochrome oxidase, all according to standard methods.^16^ Additionally, sections (8 μm) were immunohistochemically labelled with monoclonal antibodies to type 1 fibre slow myosin heavy chain (MAB1628, clone NOQ7.5.4D, Chemicon, diluted 1:50 in PBS) and type 2A fibre myosin heavy chain (A4.74, DSHB, diluted 1:5 in PBS). Labelled fibres were visualised using biotinylated sheep anti‐mouse IgG (RPN1001, Cytiva, diluted 1 in 200 in PBS), streptavidin‐HRP (016‐030‐084, Jackson Immunoresearch, diluted 1:500 in PBS) and ImmPACT DAB substrate (Vector Laboratories, SK‐4105, prepared according to manufacturer's instructions). Type 2X fibres were identified by exclusion. Histopathological evaluation was conducted by a single author (RP) who was unblinded to other clinical details.

### 2.4 Data analysis

Statistical analyses were performed using SigmaPlot15 (Systat Software, San Jose, CA, USA). The horses were divided into two groups based on muscle histopathology, Group M with histopathological changes consistent with a chronic idiopathic myopathy, and Group N with normal findings in their muscle biopsy samples. Data were not normally distributed, and descriptive statistics are therefore presented as median and 25th and 75th quartiles. Due to the low number of cases and the descriptive nature of the study, only unpaired *t*‐test (Mann–Whitney Rank Sum test) on the ranks was performed comparing baseline serum CK and AST activities between the groups, and comparing baseline CK with CK activities 4–6 and 24 h after an exercise test. A *p*‐value below 0.05 was considered significant.

## 3 RESULTS

### 3.1 Clinical signs

Twelve horses had histopathological changes in muscle biopsy samples (hence assigned to Group M), while five horses (Group N) had muscle that was considered histologically normal. Phenotypic traits presented in both groups included tachypnoea/laboured breathing both during exercise (3 horses) and at rest (3 horses), coughing (4 horses), fatigue after mild exercise (5 horses), reluctance to tölt or canter (6 horses) and stumbling during riding (4 horses). None of the horses had overt ataxia or muscle atrophy. In Group M, one horse was reported to lie down frequently, and one dragged the pelvic limb toes bilaterally. In Group N, one horse was reluctant to go forward during riding, and one repeatedly stood as if to urinate under saddle.

### 3.2 Additional diagnostics

Additional diagnostics and the resulting diagnoses for the included cases and controls are presented in Table 2. Four horses had more than one additional diagnosis.

**TABLE 2:** Concurrent diagnoses for the included horses, all with the possibility to affect performance.

	Group M (*n* = 12)	Group N (*n* = 5)
Low grade multi‐limb lameness	5	1
Mild–moderate equine asthma	5	1
Dorsal dislocation soft palate	2	0
Equine gastric ulcer syndrome	1	1
Hepatic disease	1	0

### 3.3 Muscle enzyme activities

Serum CK and AST activities are presented in Table 1. Median baseline CK activities were slightly increased in both groups (reference value <348 U/L), with no significant difference observed between the groups (*p* = 0.2). Median AST values were increased in Group M (reference value <400 U/L) whereas Group N had a median of 400 U/L. This difference was also not significant between the groups (*p* = 0.2). In 4/10 horses tested in Group M, CK activities increased more than threefold 4–6 h post‐exercise, whereas no increases were observed in the three horses tested in Group N. Significant differences in CK activities between groups were identified 4–6 h post‐exercise (*p* = 0.02) and at 24 h post‐exercise (*p* = 0.02). A significant difference was found for CK activities between baseline and 4–6 hrs (*p* = 0.03), but not between baseline and 24 h (*p* = 0.4) in Group M.

### 3.4 Histopathological findings

**FIGURE 1:**
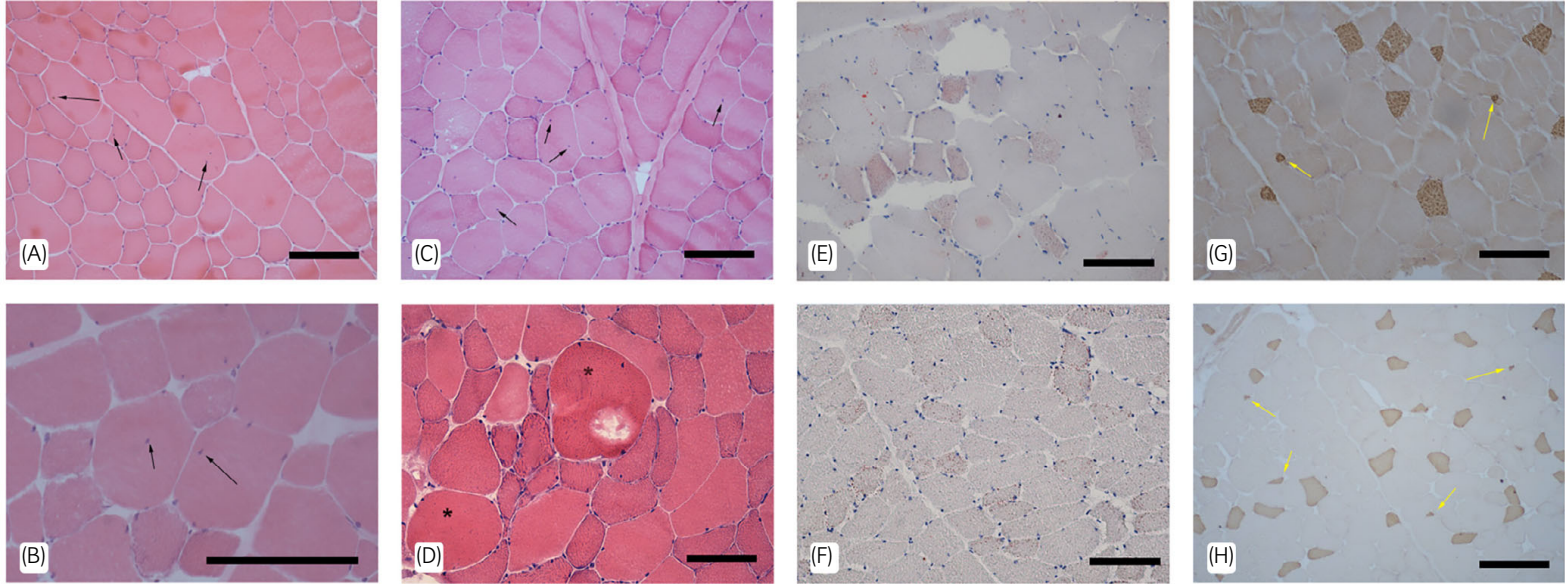
Examples of histopathological changes in muscle biopsy samples from Icelandic horses with poor performance. Internalised nuclei (generally indicative of muscle regeneration) (A–C; black arrows). Fibre hypercontraction and hypertrophy (D; *). Endoplasmic and sarcoplasmic lipid accumulation (E, F; orange spots/drops). Atrophied/hypotrophic type 1 (slow twitch) muscle fibres (G, F; yellow arrows). A–D—haematoxylin and eosin; E, F—oil red O; G, H—immunohistochemistry for type 1 fibre myosin heavy chain (brown fibres are type 1). Scale bars: all 100 μm.

Histopathological examination of Group M revealed in all cases a chronic, idiopathic myopathy ranging from mild to severe characterised by non‐specific features of both muscle degeneration and regeneration. One horse had dense, but normally appearing sarcoplasmic glycogen, but no other glycogen storage abnormalities or myofibrillar defects were detected in any of the samples. Muscle fibre morphology was generally normal, though fibre size variation was present (subjectively) in 6/12 horses. Several horses had isolated and occasional hypercontracted and hypertrophied fibres. No evidence of inflammation or excessive endomyseal fibrosis was found. 8/12 horses in Group M exhibited a few (1–2 per x10 field) to substantial (>5) internalised nuclei while the remaining 4/12 exhibited only occasional (<5 per 5 mm diameter section) internalised nuclei. In contrast, horses in Group N had very few or no internalised nuclei and lacked other non‐specific myopathic features. Several horses (5/12) demonstrated hypotrophy or atrophy of oxidative fibre types or prominent oxidative fibre size variation and sarcoplasmic lipid accumulation; in contrast, horses in Group N had only occasional oxidative fibres with lipid droplets. Mitochondrial histochemistry and fibre typing immunohistochemistry yielded normal findings across all horses. Representative histopathological changes can be seen in Figure 1.

## 4 DISCUSSION

With this case series, we introduce a novel myopathy syndrome in Icelandic horses presented with poor performance. While an aetiology (or aetiologies) has not yet been established, an underlying genetic predisposition cannot be excluded. To date, ocular,^17^ neurological^18^ and dermatological^19^ diseases in the Icelandic breed with suspicion of a genetic component have been reported, and the Icelandic breed is particularly susceptible to inbreeding due to the combination of years of isolation in Iceland and, subsequently, its growing global popularity.^20^

### 4.1 Clinical presentation and serum CK activity

The clinical presentation of nonspecific poor performance encompassed various additional historical signs including stumbling during riding, stiff gait and reluctance to perform the tölt. These clinical signs have also been associated with other conditions, including equine gastric ulcer syndrome, equine asthma and endocrinopathic laminitis.^14^, ^21^, ^22^ In this study, it was not possible to fully elucidate whether the poor performance observed in these horses was primarily related to muscle pain or weakness, respiratory issues, an underlying orthopaedic problem or a combination of these factors. To better differentiate clinical signs attributed to myopathy, future studies should include a larger sample size and a control group without impaired performance in order to draw more reliable conclusions.

Equine asthma and multi‐limb lameness were common additional findings in the studied horses. In Denmark, Icelandic horses have a prevalence of mild to severe equine asthma of 44%, depending on their housing conditions, despite owners reporting no signs of coughing or poor performance.^23^ Based on these findings, it is possible that the equine asthma identified in five cases in the current study could fully or partially account for their decreased performance; however, asthma might instead have been an incidental finding. Multi‐limb lameness might reflect the clinical presentation of Icelandic horses with idiopathic myopathy, although not consistently noted or examined in all cases. In a recent study, all examined Icelandic horses were found to be lame, with most exhibiting multi‐limb lameness.^12^ The cause of lameness was not determined, and an underlying myopathy might have been present in some of the horses.

The most commonly diagnosed myopathies in other breeds are associated with exercise and include recurrent exertional rhabdomyolysis (RER), polysaccharide storage myopathy type 1,^7^, ^24^, ^25^ and exercise‐associated myopathy syndrome (EAMS).^26^ Although not statistically significantly different between groups, the horses diagnosed with idiopathic myopathy had increased baseline muscle enzyme activities (CK and AST). Baseline CK measurements in both groups might have been affected by other factors that were not controlled, such as recent transportation.^27^ A 2–3‐fold increase in CK activity after exercise is consistent with EAMS. In the present study, one‐third of the horses in Group M had baseline CK activities >1000 U/L, suggesting ongoing, chronic myopathy. Additionally, one‐third of these horses experienced more than a threefold increase in CK activity 4–6 h post‐exercise, a pattern not observed in any horses from Group N. Furthermore, a significant difference in CK activities between the two groups was identified at 4–6 and 24 h post‐exercise, despite the small sample size. The increased CK activity in some horses in Group M suggests the possibility of a generalised myopathy that might only be detectable with exercise when it manifests as poor performance. Due to the nature and small sample size in the current study, it was not possible to explore any relationship between the degree or type of histopathological changes and the CK activity response to exercise.

### 4.2 Histopathological findings

Histopathological findings in these myopathic horses were generally nonspecific, variable and characterised by evidence of degeneration and regeneration of muscles, typified by the presence of internalised nuclei in some muscle fibres and variation in fibre size. Sarcoplasmic lipid accumulation, seen in several horses in Group M, is associated with lipid storage myopathies, and in horses is recognised in the often fatal, acquired multiple acyl‐coA dehydrogenase deficiency related to *Acer pseudoplatanus* (Sycamore)‐associated hypoglycin A toxicity (atypical myopathy).^28^ Horses with atypical myopathy exhibit no clinical similarity to the chronic idiopathic myopathy recognised in this study, and none of the included horses had a history of exposure to Sycamore material. Nonetheless, a variety of other (acquired and inherited) lipid storage myopathies are reported in other species, including other forms of multiple acyl‐coA dehydrogenase deficiency.^29^, ^30^ Furthermore, a possible lipid storage myopathy has been described in a paint foal.^31^

### 4.3 Limitations

This case series has several limitations. By design, it is a descriptive study, with the sole inclusion criterion being muscle biopsy in a specific breed presenting with poor performance. In most cases, biopsies were obtained due to the suspicion of an underlying myopathy associated with increased CK activity and/or mild multi‐limb lameness; however, in some instances, muscle biopsy was performed solely on the basis of reduced performance. A notable limitation is the absence of a control group of horses with normal performance. Also, the horses did not have the same diagnostic procedures for identifying additional diseases. Baseline serum CK and AST activities were not measured at specified time points and might have been influenced by outside factors. Furthermore, only muscle biopsies of semimembranosus were examined: other muscles might have provided additional information. Finally, because histopathological assessment was conducted as part of diagnostic evaluations, the pathologist was given access to clinical details; such evaluation, while increasing the positive predictive value, might have introduced bias.

In conclusion, the combination of breed‐specific traits, phenotypic characteristics and observed histopathological muscle changes does not align with previously documented myopathies in the Icelandic horse. Future research should involve a prospective, controlled study including a larger sample size of both affected and unaffected Icelandic horses. Additionally, this study underscores the importance of considering underlying myopathy in Icelandic horses exhibiting decreased performance, multi‐limb lameness, with normal and/or increased serum creatine kinase (CK) activity at rest or post‐exercise.

## FUNDING INFORMATION

This study was supported by The Danish Levy Foundation.

## CONFLICT OF INTEREST STATEMENT

The authors declare no conflicts of interest.

## AUTHOR CONTRIBUTIONS

**Sanni Hansen:** Conceptualization; investigation; funding acquisition; writing – original draft; methodology; writing – review and editing; software; formal analysis; project administration; data curation; supervision; resources. **Charlotte Hopster‐Iversen:** Conceptualization; investigation; funding acquisition; writing – review and editing; methodology; supervision. **Lise Berg:** Conceptualization; investigation; funding acquisition; writing – review and editing. **Julie Fjeldborg:** Conceptualization; investigation; funding acquisition; writing – review and editing. **Claire Massey:** Writing – review and editing; formal analysis; data curation; methodology; conceptualization. **Richard J. Piercy:** Conceptualization; investigation; writing – review and editing; methodology; formal analysis; supervision; data curation; resources. **Helena Carstensen:** Conceptualization; investigation; writing – original draft; methodology; writing – review and editing; formal analysis; data curation.

## DATA INTEGRITY STATEMENT

Sanni Hansen, Richard Piercy and Helena Carstensen had full access to all the data in the study and take responsibility for the integrity of the data and the accuracy of the data analysis.

## ETHICAL ANIMAL RESEARCH

Ethical approval for the study was granted by the Ethical Committee at the University of Copenhagen Large Animal Teaching Hospital (2021‐015).

## INFORMED CONSENT

Explicit owner consent for inclusion of animals in this study was not obtained. All owners/trainers were made aware that case information may be used for research in general.

## PEER REVIEW

The peer review history for this article is available at https://www.webofscience.com/api/gateway/wos/peer-review/10.1111/evj.14519.

## Data Availability

The data that support the findings of this study are openly available in Mendeley Data at https://doi.org/10.17632/gfrhzyy9v5.1.
